# Recombinant Vaccine–Derived Poliovirus in Madagascar

**DOI:** 10.3201/eid0907.020692

**Published:** 2003-07

**Authors:** Dominique Rousset, Mala Rakoto-Andrianarivelo, Richter Razafindratsimandresy, Bakolalalo Randriamanalina, Sophie Guillot, Jean Balanant, Philippe Mauclère, Francis Delpeyroux

**Affiliations:** * Institut Pasteur de Madagascar, Antananarivo, Republic of Madagascar; † Ministry of Health, Antananarivo, Republic of Madagascar; ‡ Institut Pasteur, Paris, France

**To the Editor:** Between October 2001 and April 2002, five cases of acute flaccid paralysis associated with vaccine-derived poliovirus (VDPV) type 2 isolates were reported in the southern province of the Republic of Madagascar. The first patient, an 11-year-old child from the urban district of Toliara, first experienced paralysis on October 29, 2001. Three other children, 6, 9, and 14 months of age from Ebakika village, in a rural district of Taolagnaro (250 miles east of Toliara), showed signs of poliomyelitis between March 21 and March 26, 2002. The last case-patient, a 20-month-old child from Ambanihazo village (6 miles north of Ebakika), came into contact with one of the three case-patients in Ebakika in March 2002, and symptoms developed on April 12, 2002 (*1*). None of the patients had been fully vaccinated against poliomyelitis.

Nine type 2 poliovirus (PV) strains were isolated. A restriction fragment length polymorphism (RFLP) assay, with three different genomic regions amplified by reverse transcription–polymerase chain reaction (RT-PCR) and four different restriction enzymes (*Hin*fI, *Dpn*II, *Rsa*I, and *Dde*I) were used to characterize the PV isolates at the molecular level (*2*). The RFLP profiles of all of the isolates in the two capsid protein regions were identical to that of the type 2 strain of the oral polio vaccine (OPV) in the VP1-2A region (nucleotides 2,872 to 3,647) but slightly different in the VP3-VP1 region (nucleotides 1,915 to 2,883). The observed differences allowed us to distinguish two groups (isolates from Toliara and isolates from Taolagnaro) and two subgroups (isolates from March and isolates from April). The RFLP profiles of isolates in the noncapsid region, at the 3′-terminal end of the genome (polymerase 3D and 3′ noncoding regions: nucleotides 6,535 to 7,439) also confirmed the presence of two separate groups. These last profiles were completely different from those of the three reference vaccine strains, suggesting recombination with other enteroviruses.

Partial genomic sequencing confirmed these observations. The entire VP1 region (903 nucleotides) of the type 2 PV strains from Toliara and Taolagnaro differed from the type 2 OPV strain by 1% and 2.5% nucleotides, respectively. This difference may indicate that the two strains had been multiplying or circulating for approximately 1 and 2.5 years, respectively. Taolagnaro strains are closely related to each other (<1% nucleotide difference) but appear to be very different from Toliara strains (2.9% nucleotide difference), indicating the existence of two genetic lineages. The sequencing of the noncapsid region (440 nucleotides corresponding to nucleotide positions 6,705 to 7,144 of the Sabin 2 genome) confirms the existence of two lineages derived from different recombination events with two nonidentified enteroviruses of the phylogenetic cluster C. This cluster, based on sequence similarity, includes some coxsackieviruses and all PV strains (*3*).

We tried to identify the donor strains for sequences in the 3' terminal end of these recombinant strains by aligning the nonidentified sequences with homologous enterovirus sequences available in a nucleotide sequence database (FASTA, version 3.3 applied to GenBank) (*4*). The highest percentages of nucleotide sequence identity were those with PVs and with most other cluster C enteroviruses available in the database (87% to 91% nucleotide identities). No wild PV strains have been isolated in Madagascar since 1997 despite surveillance and investigation of viral causes of acute flaccid paralysis cases (*5*). Thus, that the detected VDPVs were the product of recombination between OPV strains and two nonpolio enteroviruses is more likely than that they were the product of OPV strains and two different undetected wild PV strains. However, we cannot exclude the possibility that wild PVs were imported or circulating silently for a while.

In response to the outbreak, the local health authorities conducted house-to-house vaccination with OPV. Further field investigations were carried out to determine the extent to which VDPV had spread and to search actively for other cases. Data analysis is in progress.

As with the other epidemics in Egypt and Hispaniola, VDPV circulated in a province of Madagascar with low OPV coverage (*6*,*7*). Because a high OPV coverage rate helps prevent the circulation of both VDPVs and wild PVs, obtaining and maintaining high rates of immunization coverage are essential (*8*). Moreover, two recombinant VDPV lineages in Madagascar indicate that recombination is frequent between OPV and cluster C enteroviruses. Similar recombinant VDPVs have been implicated in the epidemics in Hispaniola and in the Philippines (*6*,*9*). Determining whether the neurovirulence and transmissibility of these VDPVs could be the result of the recombination with nonpolio enteroviruses is important. These VDPVs have major implications for the cessation of immunization with OPV after certification that wild PV has been eradicated.
